# LncRNA-mRNA co-expression analysis revealed 8 core lncRNAs in rheumatoid arthritis of collagen-induced arthritis rats

**DOI:** 10.1186/s12920-022-01398-3

**Published:** 2022-11-26

**Authors:** Yuqi Wen, Cailin He, Yang Wang, Siqin Zeng, Bo Yang, Xingui Xiong

**Affiliations:** National Clinical Research Center for Geriatric Disorders, Xiangya Hospital Central South University, Changsha, 410008 People’s Republic of China

**Keywords:** Rheumatoid arthritis, Long non-coding RNA, Microarray

## Abstract

**Backgrounds:**

Rheumatoid arthritis (RA) is a chronic inflammatory and autoimmune disease. Current studies suggest that long noncoding RNAs (lncRNAs) may be key regulators in pathogenesis.

**Methods:**

Analyzed lncRNAs and mRNAs using microarrays to find key differentially expressed lncRNAs in RA. GO and KEGG enrichment analysis together with coding non-coding co-expression (CNC) network was used for comprehensive analysis. Verify that their expression levels are consistent with the chip results by qRT-PCR.

**Results:**

There are 268 differentially expressed lncRNAs (DELs) and 286 differentially expressed mRNAs (DEMs). We found 8 core lncRNAs through the CNC network. Eight highly significantly differentially expressed lncRNAs corrected with microarray profiles. The functions and associated pathways of significantly differentially expressed lncRNAs were predicted by GO and KEGG analysis. They may be involved in the pathogenesis of RA.

**Conclusion:**

The differential expression profiles of lncRNAs and mRNAs in the collagen-induced arthritis rat model preliminarily predicted functions through comprehensive analysis. However, its exact role and specific mechanism remain to be further studied.

**Supplementary Information:**

The online version contains supplementary material available at 10.1186/s12920-022-01398-3.

## Introduction

Rheumatoid arthritis (RA) is a common systemic inflammatory and autoimmune disease [1]. The disease is endemic worldwide, affecting approximately 2.5 out of every 1000 adults [2]. RA is a chronic progressive disease that can gradually lead to irreversible joint damage, motor dysfunction and even lifelong disability [3]. When the cost of medicines becomes a major component of direct costs, there is a heavy financial burden on individuals and society [3]. There are a variety of medication regimens, including non-steroidal anti-inflammatory drugs (NSADs), disease-modifying antirheumatic drugs (DMARDs), etc. [4]. However, current treatment options do not permanently cure RA.

The pathological progression of RA can facilitate clinical treatment. The current study also revealed that long noncoding RNAs may be involved in pathogenesis [5]. Long non-coding RNAs (lncRNAs) are longer than 200 nucleotides and lack protein-coding capacity [6]. They were previously thought to be 'transcriptional noise', but emerging evidence suggests that lncRNAs may have potential functions [7, 8]. Studies have shown that the following lncRNAs (such as HOTAIR, PVT1, MALAT1, ZFAS1, etc.) may be key regulators of RA [9–12]. The functions and mechanisms of lncRNAs in RA remain unclear [7]. Expression profiling is the first step in obtaining differentially expressed lncRNAs (DELs) and mRNAs (DEMs). We discover novel important lncRNAs and predicted their potential functions by expression profiling.

## Material and methods

### Animal model and sample preparation

Male Sprague Dawley rats (n = 10) aged 7 to 8 weeks were selected for this experiment. All rats were housed in the same way in the Department of Laboratory Zoology, Xiangya School of Medicine, Central South University. Group T was the CIA rat model group, and group C was the blank control group. Arthritis was induced by subcutaneous injection of 50ul emulsified CII at the base of tail of 5 rats in group T. The emulsion was prepared by mixing bovine type II collagen and complete Freund's adjuvant (Chondrex, USA) 1:1. A booster injection with bovine type II collagen and incomplete Freund's adjuvant was added after 7 days [13, 14]. Arthritis severity was monitored after 21 days of feeding by using a macroscopic scoring system [15]. After euthanasia, synovial tissue was removed from each rat.

### RNA extraction and quality control

Total RNA was extracted from synovial tissue samples using TRIzol reagent (Invitrogen life technologies, USA). RNA concentration and purity were measured by NanoDrop ND-1000 (NanoDrop Technologies, USA). The integrity of RNA and DNA contamination was assessed by denaturing agarose gel electrophoresis (Invitrogen life technologies, USA).

### Sample labeling and array hybridization

Arraystar Rat LncRNA Arrays V3 are used to analyze lncRNAs as well as complete sets of protein-coding mRNAs. RNA was purified from total RNA after removal of rRNA using the kit (mRNA-ONLY™ Eukaryotic mRNA Isolation Kit, Epicentre, USA) according to the manufacturer's instructions. The mRNA was then amplified and transcribed into fluorescent cRNA along the entire length of the transcript using a random priming method (Arraystar Flash RNA Labeling Kit, Arraystar). Labeled cRNA was purified by RNeasy Mini Kit (Qiagen, Germany). The hybridization arrays were finally washed, then mounted and scanned with an Agilent DNA Microarray Scanner (P/N G2505C).

### GO and KEGG pathway analysis

Gene Ontology (GO) enrichment analysis is a taxonomic entry point for gene function. It includes three sub-items: Molecular Function (MF), Cellular Composition (CC), and Biological Process (BP). We used GO (http://www.geneontology.org) to classify differentially expressed mRNAs into three categories based on enrichment scores and P-values (*p* < 0.05) [16, 17]. With reference to the latest KEGG (http://www.genome.jp/kegg/) biological pathways in database entries, Fisher's exact test and X2 test were used to select significant pathways. Significant thresholds are defined by p-values (*p* < 0.05) [18].

### Quantitative real-time PCR validation

A reagent—2X PCR master mix (Arraystar, USA) was used according to the instructions of the QuantStudio5 Real-time PCR System (Applied Biosystems, USA). Primer 5.0 was used for primer design, and the primers used are shown in Table 1. The raw data were normalized to the expression of β-actin to obtain the relative expression of target lncRNAs.

**Table 1:** The sequences of primers used for qRT-PRC

Gene symbol	Forward primer (5′ to 3′)	Reverse primer (5′ to 3′)
XR_338924	CTGCAAAGAGTGTGAAAATGC	GCCGACTTCAGGCACATAA
ENSRNOT00000078133	ACCAGGACCGCCCATAAATC	AAACCTTCAACAGTTTGACTGAA
ENSRNOT00000077292	GTGGGTTCCAGTTGATGACA	GGCATTGAATCCCATTACAG
ENSRNOT00000077294	TTCACCACCTACCTTCAGATTC	GCCCACCAACTAACCAACAA
uc.10-	TTCATGTCAAACCGCACTTAAT	TGAGTGTAGAGGAGCAGAGGC
NR_132636	AAACTGAACAAAACCTCGCC	GGTCTCTCTTCTCTCCCCTGCT
XR_146333	GCCTGAGTGAGTGACAGAATACC	TGTGATCCCAACCAGCCG
ENSRNOT00000077718	ACCTCTGACATCTTCCTTGAGA	GCTCTTCAGCATCCCTTACTAC

### Construction of coding-noncoding co-expression networks

To further discover the relationship between differentially expressed lncRNAs and mRNAs, we constructed a coding-non-coding co-expression (CNC) network by Cytoscape 3.8.2. By calculating the Pearson correlation coefficient (PCC), we selected gene pairs with |PCC|> 0.97 and *p* < 0.05, and then imported their data for network construction.

### Statistic analysis

An independent samples t-test was used to screen for DEL and DEM. Thresholds were set: FC > 2 or FC < 0.5 (fold change, absolute ratio of normalized intensities between two conditions), *p* < 0.05 was expressed as statistical difference (SPSS 17.0 software).

## Results

### Quality assessment of LncRNA and mRNA expression levels in CIA models

**Fig. 1:**
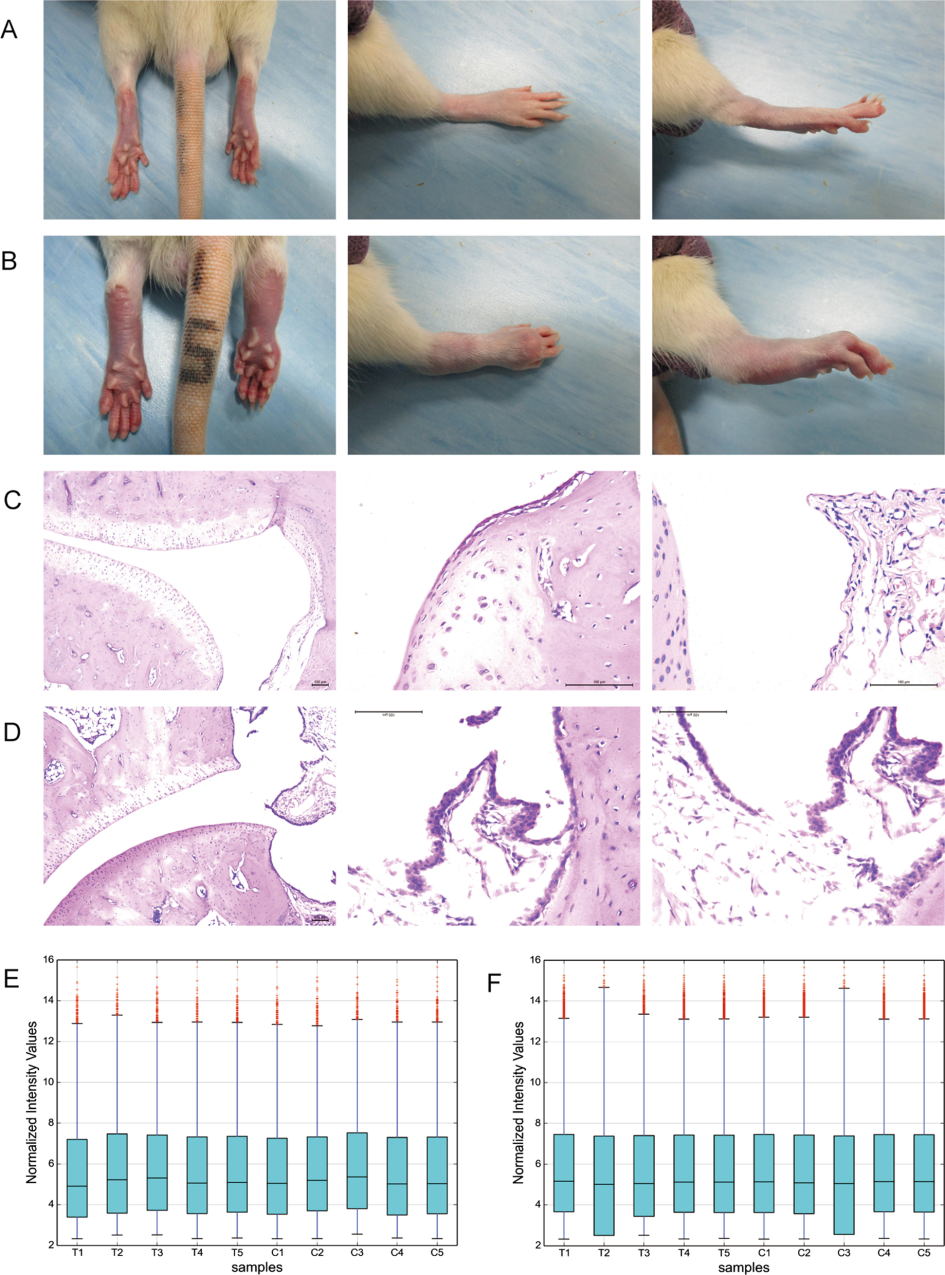
Quality assessment of LncRNA and mRNA expression levels in the CIA model. Appearance and HE-stained sections of ankle joints of **A**, **C** normal rats and **B**, **D** CIA rat models. Normalized intensities of **E** lncRNA and **F** mRNA

We utilize the collagen-induced arthritis (CIA) rat model to study DELs and DEMs in rheumatoid arthritis. After 21 days of modeling, the ankle joints of CIA rats showed obvious redness and swelling (Fig. 1A, B). During the experimental period, the body weight gain of the treated rats was significantly lower than that of the control group. At the same time, a significant increase in arthritis index was seen in the treated rats (see Additional file 1). Meanwhile, HE-stained sections of ankle tissue showed more inflammatory cells and tissue destruction in CIA rats (Fig. 1D) compared to normal rats (Fig. 1C). All the above evidences indicated that the CIA rat model was successfully established. The boxplots (Fig. 1E, F) show that the normalized intensity distribution is the same for all samples. DELs and DEMs were significantly up- or down-regulated between T and C groups.

### Differential expression of the lncRNAs and mRNAs

**Fig. 2:**
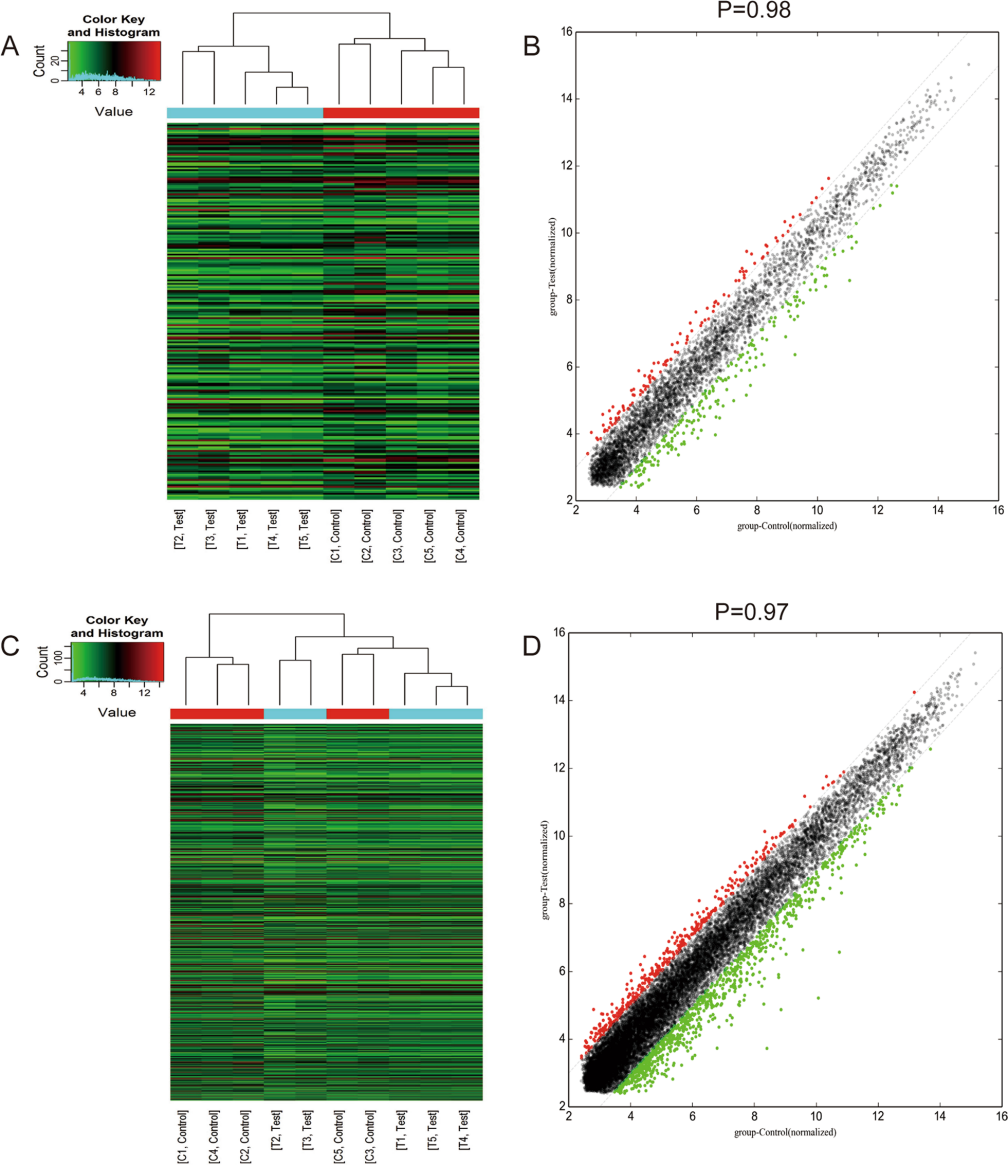
The cluster analysis of **A** DELs and **C** DEMs between C and T group. The scatter-plots of **B** DELs and **D** DEMs

Heatmaps and scatterplots (Fig. 2A–D) indicated that the differential expression trend of IncRNA and mRNA was evident. We found 268 differentially expressed lncRNAs, of which 117 lncRNAs were up-regulated and 151 lncRNAs were down-regulated. And there are 286 up-regulated mRNAs and 491 down-regulated mRNAs.

### The co-expression network of lncRNAs and mRNAs

**Fig. 3:**
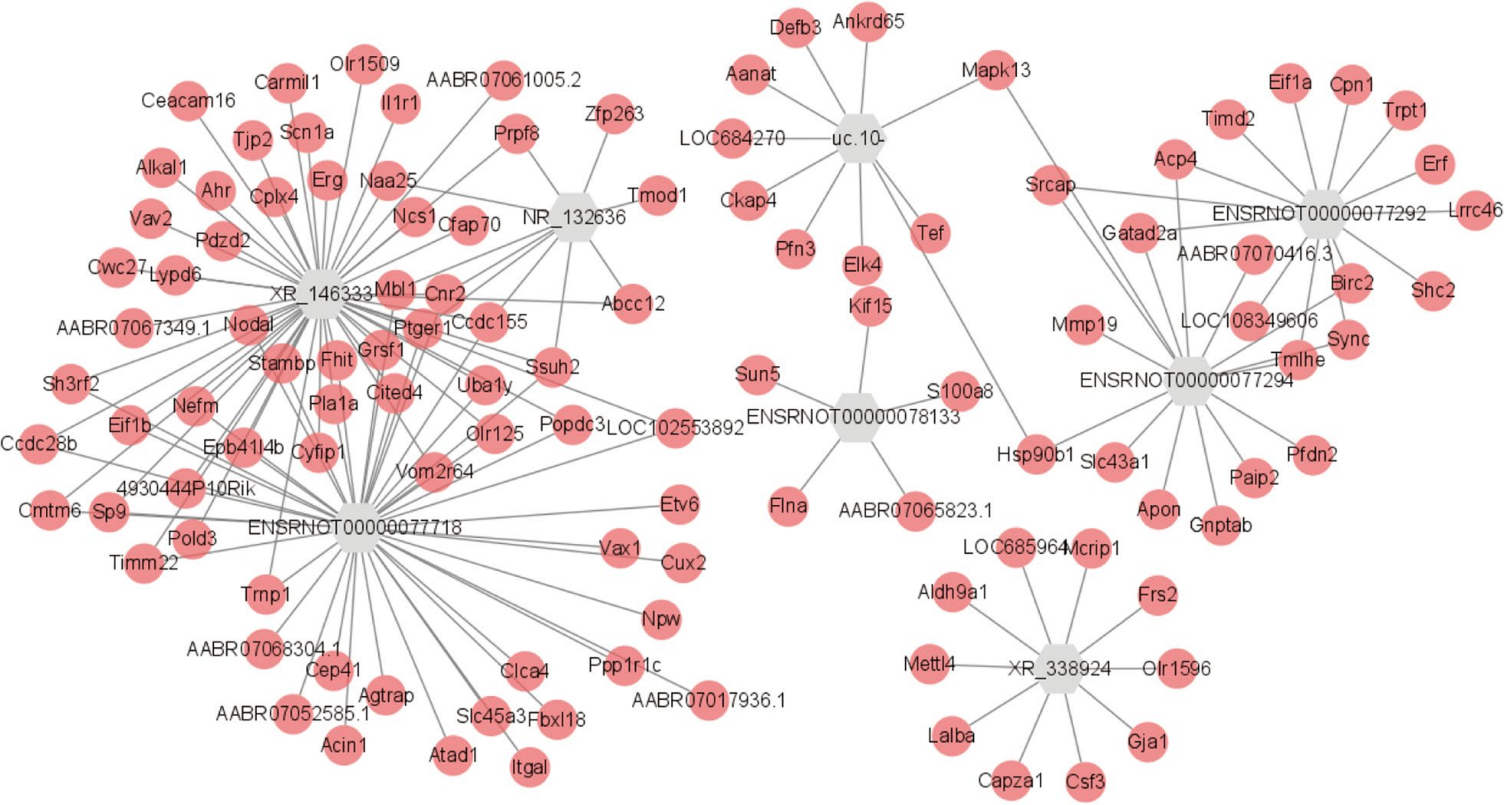
The coding non-coding co-expression (CNC) network between lncRNAs and mRNAs

The coding non-coding co-expression (CNC) network (Fig. 3) constructed between lncRNAs and mRNAs is mainly composed of 8 core lncRNAs (XR_146333, ENSRNOT00000078133, XR_338924, ENSRNOT00000077292, ENSRNOT00000077718, ENSRNOT00000077294, uc_10077294, uc_10-). Eight core lncRNAs were associated with a total of 113 mRNAs, and a total of 154 lncRNA-mRNA pairs. The expression of 8 lncRNAs was confirmed by qRT-PCR. We investigated the functions of these lncRNAs through the study of CNC networks and associated mRNAs.

### Quantitative real-time PCR validation

**Fig. 4:**
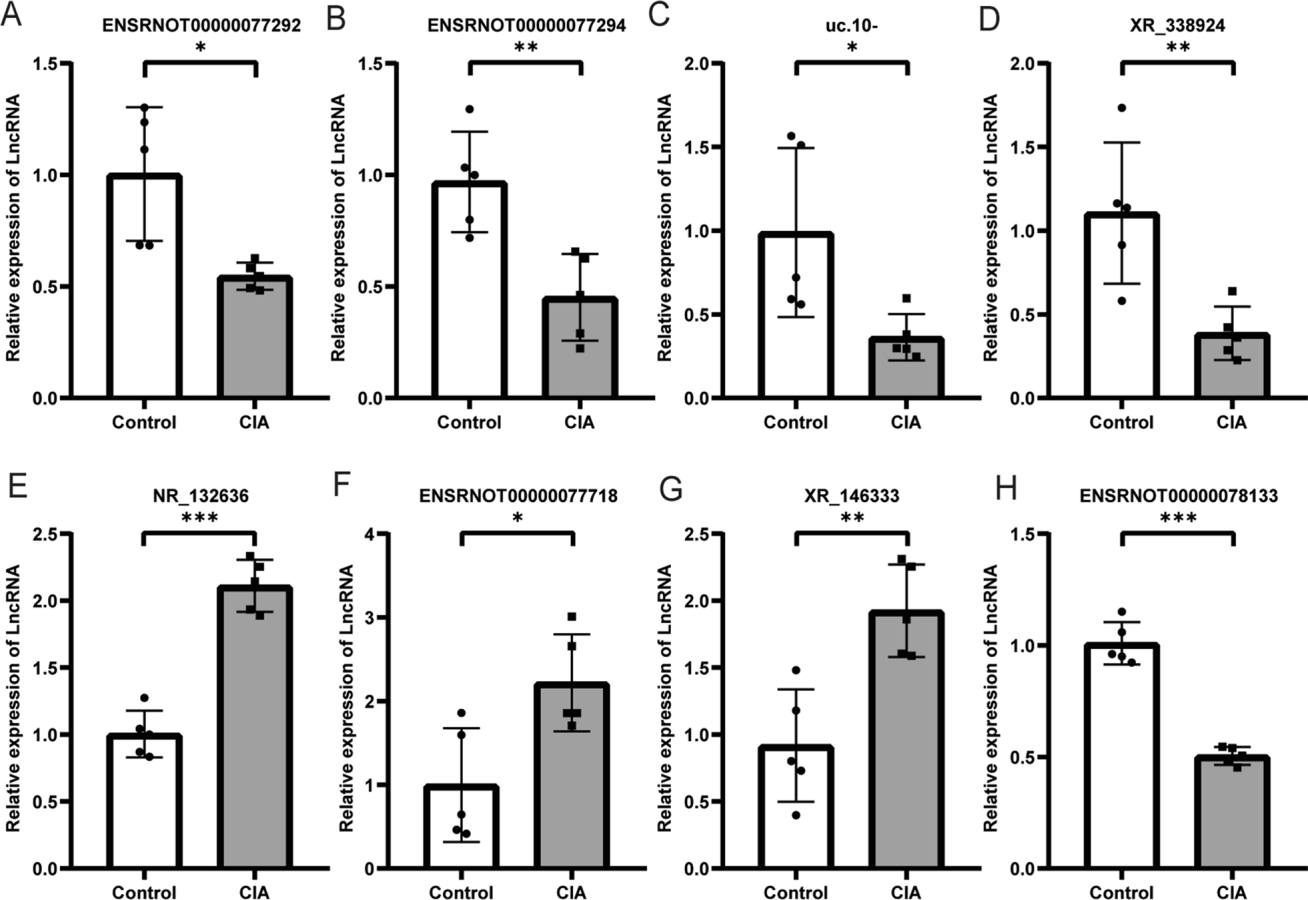
**A** Relative expression of ENSRNOT00000077292. **B** Relative expression of ENSRNOT00000077294. **C** Relative expression of uc.10-. **D** Relative expression of XR_338924. **E** Relative expression of NR_132636. **F** Relative expression of ENSRNOT00000077718. **G** Relative expression of XR_146333. **H** Relative expression of ENSRNOT00000078133

We discovered 8 core lncRNAs by CNC analysis and their expression levels were measured by qRT-PCR. There were 3 up-regulated (ENSRNOT00000077718, NR_132636, XR_146333) and 5 down-regulated (ENSRNOT00000077292, ENSRNOT00000077294, ENSRNOT00000078133, uc.10-, XR_338924) lncRNAs. Their relative expression levels were consistent with the microarray results shown in Fig. 4. It further confirmed the credibility of the microarray results.

### GO and KEGG pathway analysis of differentially expressed LncRNAs and mRNAs

**Fig. 5:**
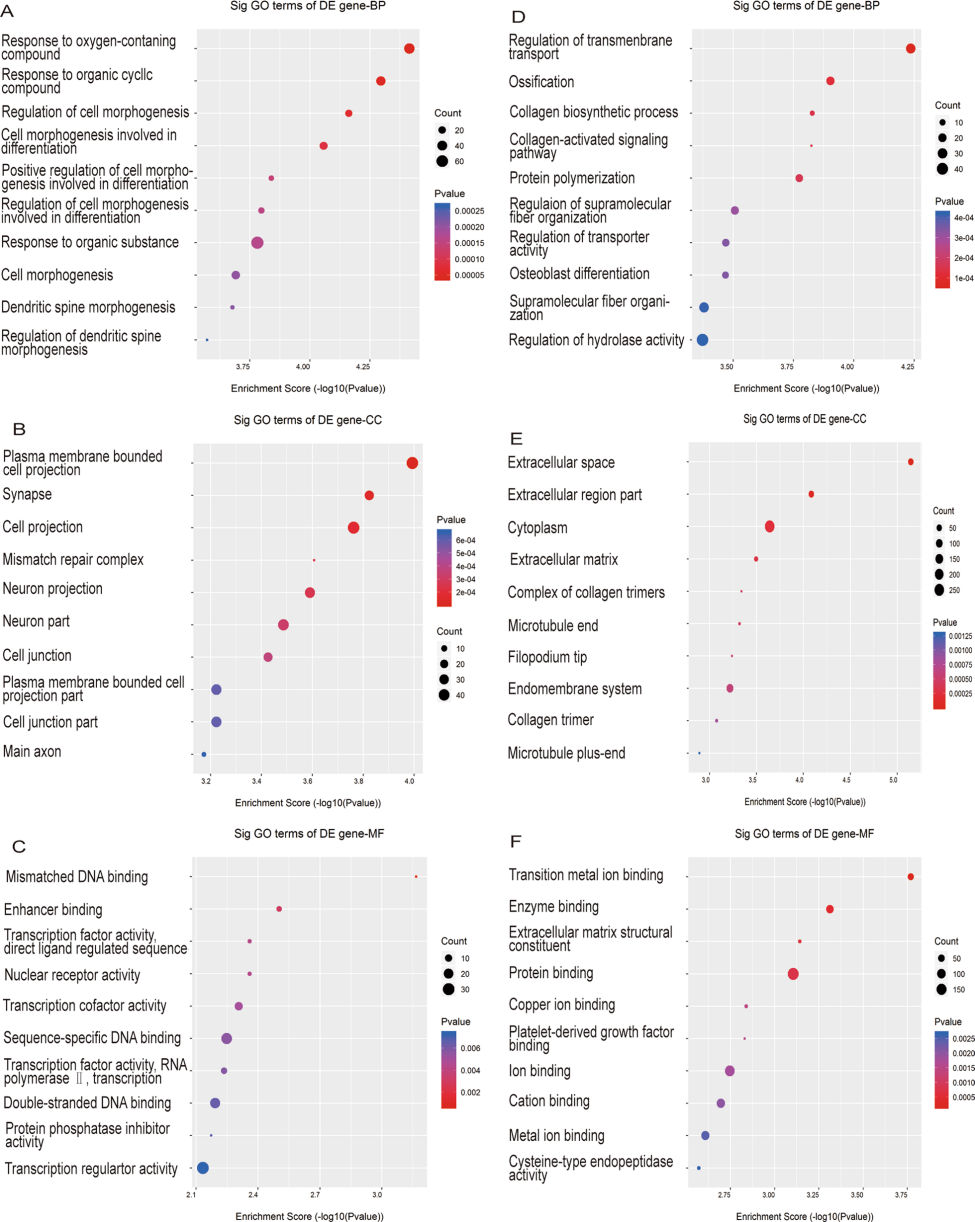
GO analysis of differentially expressed lncRNAs. The **A**–**C** up-regulated and **D**–**F** down-regulated lncRNA GO enrichment analysis

**Fig. 6:**
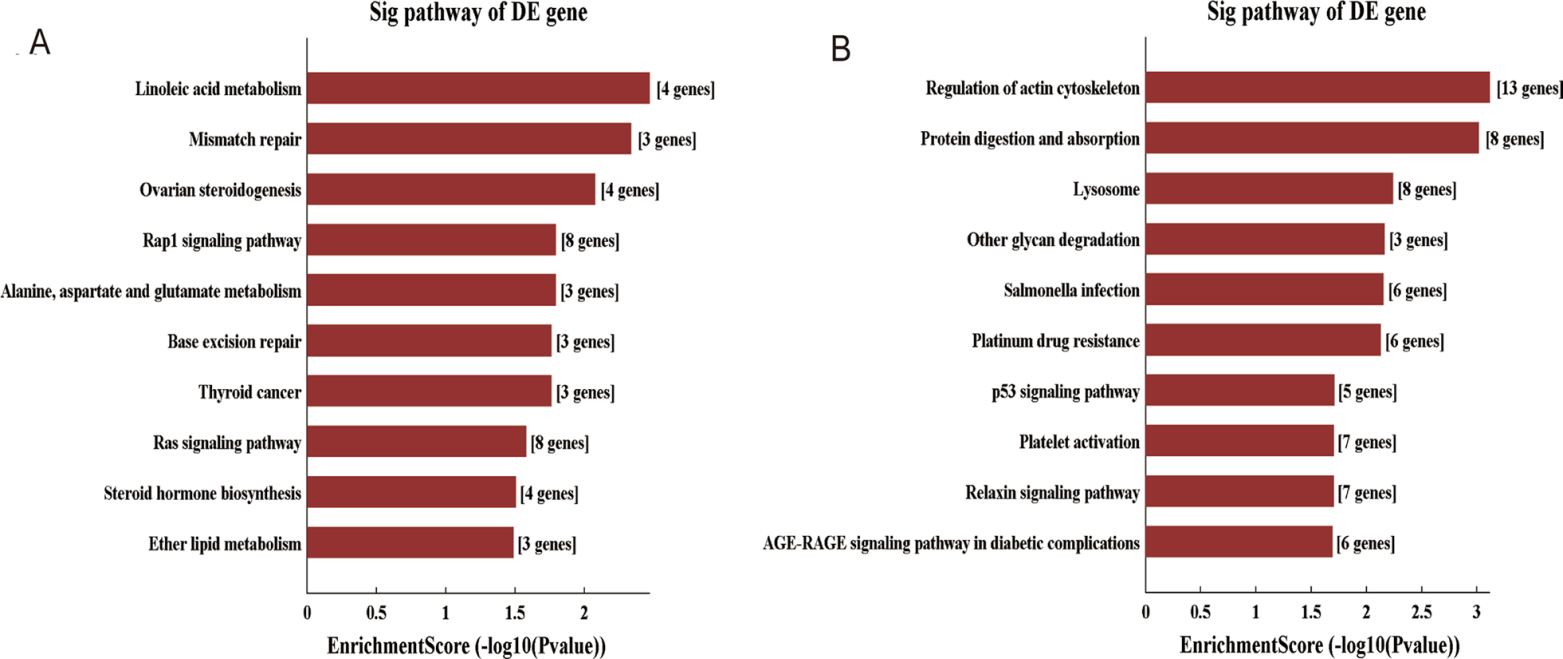
KEGG analysis of differentially expressed lncRNAs. The **A** up-regulated and **B** down-regulated lncRNA related KEGG pathway

GO analysis revealed that regulation of 'transmembrane transport', 'ossification', 'collagen biosynthetic process' and 'collagen-activated signaling pathway' were the most abundant terms among biological processes (BPs) that downregulated differentially expressed mRNAs. We also found abundant terms related to bone erosion, such as "ossification" and "osteoblast differentiation". These mRNAs structurally belong to the extracellular space in the cellular component (CC), and their molecular function (MF) is transition metal ion binding. "Response to oxygenates", "plasma membrane-bound cell projection" and "mismatched DNA binding" were the most abundant terms for up-regulated mRNAs in BP, CC and MF, respectively. KEGG pathway analysis showed that the most abundant pathways were "Regulation of the actin cytoskeleton" and "Linoleic acid metabolism" for down- or up-regulated mRNAs, respectively. The enrichment analysis results are shown in Fig. 5 and 6.

## Discussion

Rheumatoid arthritis can cause great damage to a patient's joint and motor function. Current studies have confirmed that genetic, epigenetic, environmental and other factors can interfere with its pathogenesis [1, 19]. However, the pathogenesis of RA is not fully understood. We target the pathogenesis of reverse transcription for potential biomarkers and therapeutic targets. LncRNAs are considered key regulators of chronic diseases such as RA [7, 20]. Expression profiling using microarrays is a fundamental study to discover relevant lncRNAs and speculate their functions in RA [21]. Some studies have performed transcriptome sequencing of rheumatoid arthritis patient tissues, but studies in collagen-induced arthritis rats have not been reported [22, 23]. Transcriptome research through the CIA rat model, which will lay the foundation for our future basic experiments to simulate the RA patients through rats.

In this study, we simulated the pathogenesis of RA patients through a rat CIA model. We found that 117 lncRNAs were up-regulated and 151 lncRNAs were down-regulated. And there are 286 up-regulated mRNAs and 491 down-regulated mRNAs. The results showed that RA patients had different lncRNA and mRNA expression profiles compared with healthy individuals. We selected 8 core highly differentially expressed core lncRNAs of interest to us through CNC network analysis. The results of qRT-PCR also support the reliability of high-quality microarrays.

The Gene Ontology (GO) database divides the functions of genes into three parts: cellular component (CC), molecular function (MF), and biological process (BP). Using the GO database, we can get what our target genes are mainly related to at the CC, MF and BP levels [16]. The Kyoto Encyclopedia of Genes and Genomes (KEGG) database is a database formed based on biological pathways [18]. By enrichment analysis we can predict the function of target genes and improve the efficiency of sequencing analysis. GO and KEGG pathway analysis was used for further study. Previous studies have confirmed significant enrichment for the terms "collagen biosynthetic process" and "collagen activation signaling pathway". It shows increased collagen biosynthesis in rheumatoid synovial tissue [24]. There are several types of cartilage-specific collagen, including type II (CII), type IX (CIX), type X (CIX), and type XI (CXI). CII induces the production of inflammatory cytokines on CII-reactive T cells and fibroblast-like synoviocytes (FLSs), which in turn lead to synovitis [25]. C I and CIX collagen has been found degraded in RA, leading to the degradation of rheumatoid cartilage [26, 27]. Meanwhile, collagen metabolism markers type III procollagen peptide (PIIIP) and type IV collagen 7S domain (7S-collagen) were highly observed in RA patients [28]. There were 11 mRNAs associated with differentially expressed lncRNAs that were enriched in both terms. Related lncRNAs can be selected for further study.

We constructed coding-noncoding co-expression networks to discover relationships between differentially expressed lncRNAs and mRNAs. Eight core lncRNAs are involved in the network. They are highly differentially expressed in the rat CIA model and have been validated by qRT-PCR. S100A8 is an mRNA targeted by ENSRNOT00000078133, a major leukocyte protein secreted by activated leukocytes, and binds to Toll-like receptor 4 to promote inflammation and autoimmunity. Previous studies found that S100A8/A9 was significantly elevated in serum and synovial fluid in RA patients [29]. MMP19 targeting ENSRNOT00000077294, uc.10 and ENSRNOT00000077292 was downregulated in the expression profile. It is expressed consistently with targeted lncRNAs. Previous studies have shown that MMP19 is isolated from the synovium of RA patients and may be involved in RA-related joint tissue destruction [30, 31]. The CNC network discovered the related mRNAs of 8 major lncRNAs to further reveal the functions. And through enrichment analysis, we made a preliminary bioinformatics prediction of its function.

## Conclusion

In conclusion, we found differential expression profiles of lncRNAs and mRNAs in the CIA rat model. We constructed coding-noncoding co-expression networks to discover relationships between differentially expressed lncRNAs and mRNAs. Eight core lncRNAs (XR_146333, ENSRNOT00000078133, XR_338924, ENSRNOT00000077292, ENSRNOT00000077718, ENSRNOT00000077294, uc_10077294, uc_10-) are involved in the network. Synthetic analysis is used for preliminary prediction functions. However, its exact role and specific mechanism remain to be further studied.

## Supplementary Information

**Additional file 1**. **A** Body weight of CIA rats. **B** Arthritis index of CIA rats (* *P*-value < 0.05).

## Data Availability

The datasets generated and analysed during the current study are available in the GEO repository. And the series entry could provide access to all data (GSE206097, https://www.ncbi.nlm.nih.gov/geo/query/acc.cgi?acc=GSE206097).

## Abbreviations

RA: Rheumatoid arthritis
lncRNAs: Long noncoding RNAs
CNC: Coding non-coding co-expression
DELs: Differentially expressed lncRNAs
DEMs: Differentially expressed mRNAs
NSADs: Non-steroidal anti-inflammatory drugs
DMARDs: Disease-modifying antirheumatic drugs
GO: Gene Ontology
KEGG: Kyoto Encyclopedia of Genes and Genomes
PCC: Pearson correlation coefficient
