# Supplementary figures and images for: LncRNA-mRNA co-expression analysis revealed 8 core lncRNAs in rheumatoid arthritis of collagen-induced arthritis rats
Source: BMC Med Genomics. 2022 Nov 26;15:244. doi: 10.1186/s12920-022-01398-3 (PMC9700927; doi:10.1186/s12920-022-01398-3)

**Supplementary Information**. (A) Body weight of CIA rats. (B) Arthritis index of CIA rats (* P-value < 0.05).


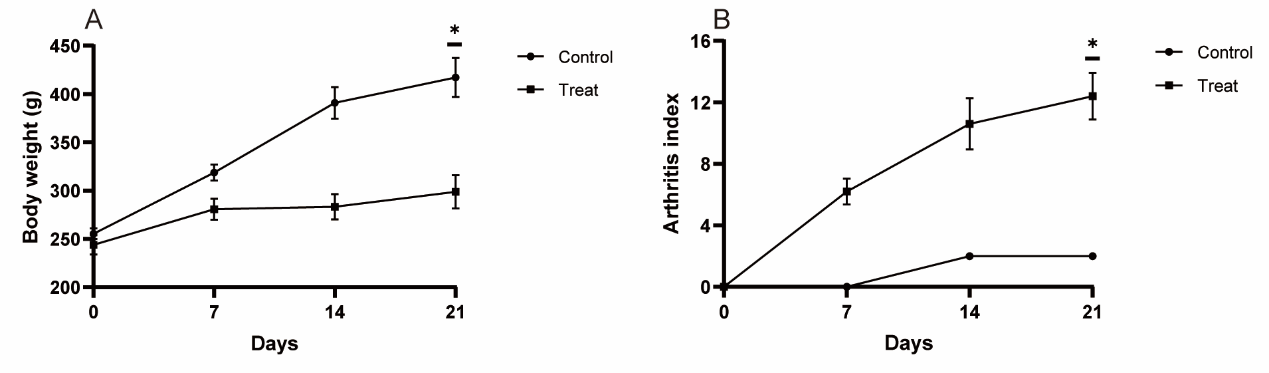

Supplement: Supplementary file 1 — Additional file 1. A Body weight of CIA rats. B Arthritis index of CIA rats (* P-value < 0.05). [file 12920_2022_1398_MOESM1_ESM.docx]
